# *MCP1 *haplotypes associated with protection from pulmonary tuberculosis

**DOI:** 10.1186/1471-2156-12-34

**Published:** 2011-04-19

**Authors:** Christopher D Intemann, Thorsten Thye, Birgit Förster, Ellis Owusu-Dabo, John Gyapong, Rolf D Horstmann, Christian G Meyer

**Affiliations:** 1 Bernhard Nocht Institute for Tropical Medicine, Dept. Molecular Medicine, Hamburg, Germany; 2 University Hospital Schleswig-Holstein, Campus Lübeck, Institute of Medical Biometry and Statistics, Lübeck, Germany; 3 Kumasi Centre for Collaborative Research in Tropical Medicine, Kumasi, Ghana; 4 College of Health Sciences, Dept. Community Health, Kwame Nkrumah University of Science and Technology, Kumasi, Ghana; 5 School of Public Health, College of Health Sciences, University of Ghana, Legon, Accra, Ghana

## Abstract

**Background:**

The monocyte chemoattractant protein 1 (MCP-1) is involved in the recruitment of lymphocytes and monocytes and their migration to sites of injury and cellular immune reactions. In a Ghanaian tuberculosis (TB) case-control study group, associations of the *MCP1 *-362C and the *MCP1 *-2581G alleles with resistance to TB were recently described. The latter association was in contrast to genetic effects previously described in study groups originating from Mexico, Korea, Peru and Zambia. This inconsistency prompted us to further investigate the *MCP1 *gene in order to determine causal variants or haplotypes genetically and functionally.

**Results:**

A 14 base-pair deletion in the first *MCP1 *intron, int1del554-567, was strongly associated with protection against pulmonary TB (OR = 0.84, CI 0.77-0.92, P_corrected _= 0.00098). Compared to the wildtype combination, a haplotype comprising the -2581G and -362C promoter variants and the intronic deletion conferred an even stronger protection than did the -362C variant alone (OR = 0.78, CI 0.69-0.87, P_nominal _= 0.00002; adjusted P_global _= 0.0028). In a luciferase reporter gene assay, a significant reduction of luciferase gene expression was observed in the two constructs carrying the *MCP1 *mutations -2581 A or G plus the combination -362C and int1del554-567 compared to the wildtype haplotype (P = 0.02 and P = 0.006). The associated variants, in particular the haplotypes composed of these latter variants, result in decreased MCP-1 expression and a decreased risk of pulmonary TB.

**Conclusions:**

In addition to the results of the previous study of the Ghanaian TB case-control sample, we have now identified the haplotype combination -2581G/-362C/int1del554-567 that mediates considerably stronger protection than does the *MCP1 *-362C allele alone (OR = 0.78, CI 0.69-0.87 vs OR = 0.83, CI 0.76-0.91). Our findings in both the genetic analysis and the reporter gene study further indicate a largely negligible role of the variant at position -2581 in the Ghanaian population studied.

## Background

The monocyte chemoattractant protein 1 (MCP-1), also referred to as CCL2 (Chemokine [C-C motif] ligand 2), is a member of the small inducible gene (SIG) family. CC-chemokines are characterized by two adjacent cysteine residues close to the amino terminus of the molecule. They are involved in the recruitment of lymphocytes and monocytes and control migration of these cells to sites of cell injury and cellular immune reactions [1]. MCP-1 is produced by different cell types in response to microbial stimuli [2]. MCP-1 signals are transduced through the CCR2-receptor (chemokine [C-C motif] receptor 2). Distinct microbial components are capable to induce expression of the CCR2 receptor and to initiate, dependent on the presence of MCP-1, target-oriented roaming of monocytes.

The role of MCP-1 in tuberculosis (TB) has been subject of research since the early 1990 s. During the course of an infection with agents of the *M. tuberculosis *complex, MCP-1 is predominantly produced by CD14+ blood monocytes and by distinct alveolar epithelial cells [3,4]. Elevated plasma MCP-1 levels are found in TB patients [3], and the number of macrophages in bronchoalveolar lavage fluids in eosinophilic pneumonia correlates with plasma MCP-1 levels [5].

The gene encoding MCP-1 (*MCP1*; MIM +158105) is located in the 17q11.2-q12 chromosomal region. It consists of three exons and clusters with the loci *CCL7, CCL11, NOS2A, CCL3-5 and CCR7*. MCP-1 expression may be found in several conditions that are characterized by infiltration of mononuclear cells.

Genetic associations of *MCP1 *gene variants with susceptibility or protection against mycobacterial infection have been reported. Carriers of the *MCP1 *-2581G allele were at an increased risk of clinical TB in study groups from Mexico and Korea [4], Peru [6] and Zambia [7] compared to individuals carrying the alternative -2581A allele. For *MCP1 *-2581G, several studies have demonstrated increased gene expression *in vitro *and elevated MCP-1 plasma levels *in vivo *[4,6,8-10]. In contrast to these observations, Japanese *MCP1 *-2581AA genotype carriers exhibited higher MCP-1 plasma levels than did carriers of the -2581AG or GG genotypes [11]. While no effect of *MCP1 *-2581 variants on TB susceptibility was observed in Brazilian, Chinese, Russian and South African study groups [12-15], *MCP-1 *-2581G was associated with protection against TB in a Ghanaian case-control study group [14]. Notably, the latter finding was in clear contradiction to the findings reported in Refs. [4,6,7], where an increased TB risk was attributed to *MCP1 *-2581G carriers.

In the study of the Ghanaian TB case-control group, *MCP1 *-2581G was in weak linkage disequilibrium (LD) with another promoter variant, *MCP1 *-362C (r^2 ^= 0.27), which was even stronger associated with resistance to TB than *MCP1 *-2581G. *MCP1 *-362C has been shown to mediate increased transcriptional activity *in vitro *in a Caucasian study population [16]. Again, this finding is in contrast to the previous assumptions, namely that increased MCP-1 production might favour the occurrence of TB [4,5,8].

These partly ambiguous findings prompted us to re-examine *MCP1 *variants that might be involved in gene expression. According to the *MCP1 *haplotype structure obtained from the innate immunity website https://pharmgat.org/IIPGA2 eight variants that are located in the *MCP1 *5'-region, the first intron and in the 3'-UTR were selected and genotyped in our Ghanaian TB case-control group. Those genetic variants that showed the strongest associations with protection against TB were then subjected to a reporter luciferase gene assay in order to study gene expression.

## Results

### Alleles and genotypes

Eight *MCP1 *variants were genotyped in 2010 Ghanaian pulmonary TB cases and 2346 healthy control individuals (Table 1). P values, including those of the previous study, were corrected according to the Bonferroni-Holm procedure [17] for the eight comparisons made.

**Table 1:** Variants selected for genotyping

*MCP1*	variant	rs #	Primer oligonucleotides	Sensor/Anchor oligonucleotides
-97569	C/G	rs9891330	F-TCTGATGCAGACAGCGAG	S-GCCTCCCCCACCCCCA
			R-CACCTGGAGTCCCAGTT	A-ATAGCTGTCGGGAGAGTCTGTATTTGAAAGAGAA

-38600	C/T	rs8075337	F-CTTCTGTGAGCATTGGGT	S-ACTTCTTTTGCTGTGTTTTATTTTATTTTC
			R-AGAAACAAAAATTAGGGCATCTAC	A-CCAACATCTGGATTTCTTCAGGGACAGTTTACATT

-1727	A/T	rs3917886	F-TGGGATTCTCCAGGAAACC	S-GAAGAAGAGATACTGGAATGGAAACATCC
			R-ACCCAGCTTTCGTTAGG	A-GGGTGGGAGTCTCAGCACATCTACTATTCTGTC

Int1:96	C/G	rs41507946	F-TAAGGCCCCCTCTTCTTC	S-CCCACAGTCTTGCTTTAACGCTAC
			R-CTGTGTGGTTGGGCTCA	A-TTTCCAAGATAAGGTGACTCAGAAAAGGACAAGGG

Int1:554-567	W/D	rs3917887	F-TCCCCAGCTGATCTTCC	S-TTTAACCGCTCCTCCTTC
			R-TGACTCAGTTTCCTATGCTGTA	A-GTCCGTCTTAATGACACTTGTAGGCATTATCTAG

+1542	C/T	rs13900	F-GACCACCTGGACAAGCA	S-TAGCTTTCCCCAGACACCCTGTTTTA
			R-ATTACTTAAGGCATAATGTTTCACATC	A-CACAACCCAAGAATCTGCAGCTAACTTATTTTCCC

+2413	G/T	rs3917890	F-ATGAGACCTGAACTTATTATTTA	S-GATCATTAAGAAAGGAGAAGGAAGAGTGG
			R-TTCACCCTAACATTCAAATC	A-AGCAAATACCTGGAGGTAGAAATGGTGATGATGTGTAC

+2580	A/T	rs41343046	F-GCCCACACCAATGTCAA	S-AAGGGATTTGAATGTTAGGGTGAAAAGATA
			R-CTGAATCTCTAAACATGGCAC	A-ACTCAACTCTGTAGGTTAAAAGGAAACGTTGAGAA

F, forward primer; R, reverse primer; S, sensor; A, anchor; Int1, intron 1; D, deletion; W, wildtype

In Table 2, allelic associations of the eight newly genotyped *MCP1 *variants and of the *MCP1 *-2581 and -362 variants that were previously typed are given. The deletion of 14 bases length located in the first *MCP1 *intron, int1del554-567, was associated with protection against pulmonary TB to a similar extent as were the promoter alleles -2581G and -362C (OR = 0.85, confidence interval [CI] 0.78-0.92, P_corr _= 0.00098, OR = 0.81, CI 0.73-0.91, P_corr _= 0.0012 and OR = 0.83, CI 0.76-0.90, P_corr _= 0.00015, respectively).

**Table 2:** Allelic associations

*MCP1*	allele	cases n (frequency)	controls n (frequency)	OR	CI	**P**_**nom**_	**P**_**corr**_
-97569	C	1497 (0.38)	1745 (0.38)	1		0.99	
	G	2471 (0.62)	2889 (0.62)	1.00	[0.92-1.09]		
-38600	T	1730 (0.44)	2107 (0.46)	1			
	C	2228 (0.56)	2463 (0.54)	1.10	[1.01-1.20]	0.031	
-2581*	A	3256 (0.83)	3692 (0.80)	1			
	G	672 (0.17)	932 (0.20)	0.81	[0.73-0.91]	**0.0002**	**0.0012**
-1727	A	113 (0.03)	149 (0.03)	1			
	T	3525 (0.97)	4133 (0.97)	1.14	[0.88-1.45]	0.32	
-362*	G	2266 (0.58)	2441 (0.53)	1			
	C	1670 (0.42)	2161 (0.47)	0.83	[0.76-0.90]	**0.000019**	**0.00015**
Int1:96	C	967 (0.29)	1092 (0.26)	1			
	G	2401 (0.71)	3128 (0.74)	0.87	[0.78-0.96]	0.0055	
Int1:554-567	W	2432 (0.61)	2646 (0.57)	1			
	D	1586 (0.39)	2028 (0.43)	0.85	[0.78-0.92]	**0.00014**	**0.00098**
+1542	T	711 (0.18)	933 (0.20)	1			
	C	3291 (0.82)	3745 (0.80)	1.16	[1.04-1.29]	0.008	
+2413	T	134 (0.07)	198 (0.07)	1			
	G	1750 (0.93)	2652 (0.93)	0.97	[0.77-1.22]	0.81	
+2580	A	397 (0.10)	454 (0.10)	1			
	T	3591 (0.90)	4184 (0.90)	0.97	[0.84-1.12]	0.71	

OR, odds ratio; CI, 95% confidence interval; P values are adjusted for age, gender and ethnicity;

P_nom_, nominal P value; P_corr_, P value after Bonferroni-Holm correction; *variants -2581 and -362 were originally genotyped and described in Ref. [15]; Int1, intron 1; D, deletion; W, wildtype

**Figure 1:**
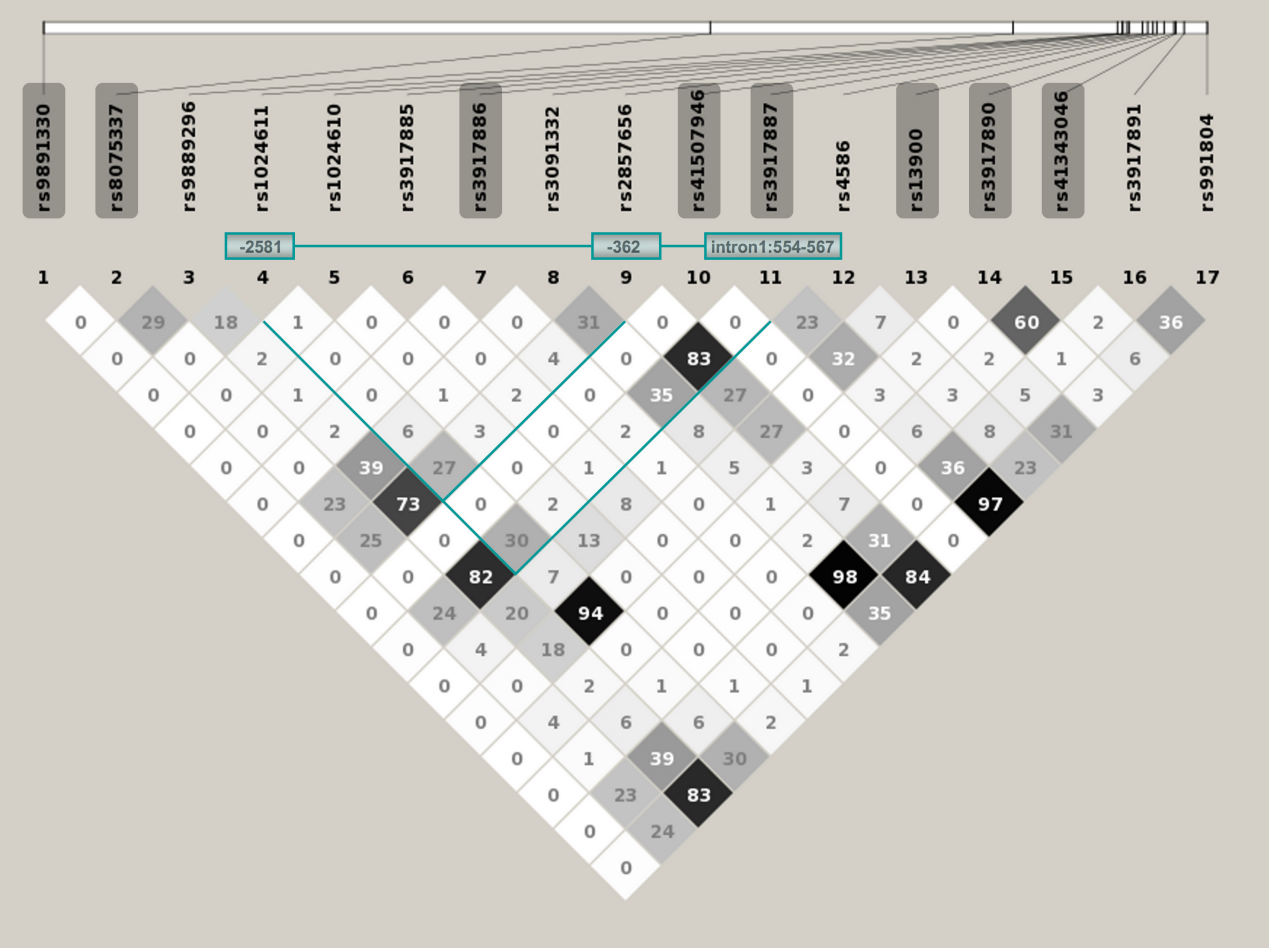
**Pairwise linkage disequilibrium (LD) plots of MCP1 variants**. Pairwise linkage disequilibrium (LD) plots of *MCP1 *variants in the present and the previous study. rs numbers of variants that were newly genotyped are shaded. The haplotype consisting of variants -2581A/G, -362C/G and int1del554-567/W are marked in turquoise blocks.

The genotype frequencies did not deviate from Hardy-Weinberg equilibrium (HWE) among cases and controls. Trend tests were performed to compare the frequencies of genotypes of cases and controls in an additive model and results were adjusted for gender, age and ethnicity. The results are given in Table 3. As also observed in the computation of allelic associations, int1del554-567 was in the trend test significantly associated with protection against TB (OR_trend _= 0.84, CI 0.77-0.92, P_corr _= 0.00098). In a genotype test where heterozygous and homozygous genotypes were individually compared to the wildtype, a strong association was seen for both heterozygous and homozygous carriers of the int1del554-567 deletion (OR = 0.80, CI 0.70-0.91, P_corr _= 0.0063 and OR = 0.73, CI 0.61-0.87, P_corr _= 0.0042, respectively), indicating a dominant genetic effect. The association of int1del554-567 was of similar strength as that in heterozygous and homozygous *MCP1 *-362C carriers in the previous study (Ref. [14]; OR_trend _= 0.83, CI 0.76-0.91, P_corr _= 0.00017). Both variants were in strong LD (r^2 ^= 0.82). int1del554-567 was also in weak LD (r^2 ^= 0.27) with the *MCP1 *promoter variant at position -2581. Figure 1 shows the r^2 ^values of pairwise LDs of all variants examined in the present and in the previous study [14].

**Table 3:** Genotype associations

*MCP1*	GT	cases n (frequency)	controls n (frequency)	OR	CI	**P**_**nom**_	**P**_**corr**_	**OR**_**trend**_	CI	**P**_**nom**_	**P**_**corr**_
-97569	CC	278 (0.14)	328 (0.14)	1				1.00	[0.92-1.10]	0.99	
	CG	941 (0.47)	1089 (0.47)	1.02	[0.85-1.23]	0.81					
	GG	765 (0.39)	900 (0.39)	1.01	[0.84-1.22]	0.92					
-38600	CC	629 (0.32)	666 (0.29)	1				0.91	[0.83-0.99]	**0.03**	
	CT	970 (0.49)	1131 (0.50)	0.91	[0.79-1.05]	0.20					
	TT	380 (0.19)	488 (0.21)	0.83	[0.70-0.99]	**0.03**					
-2581*	AA	1355 (0.69)	1472 (0.64)	1				0.81	[0.73-0.91]	**0.0003**	**0.0018**
	AG	546 (0.28)	748 (0.32)	0.79	[0.69-0.90]	**0.001**	**0.006**				
	GG	63 (0.03)	92 (0.04)	0.73	[0.53-1.02]	0.064					
-1727	TT	1708 (0.94)	1994 (0.93)	1				0.88	[0.69-1.13]	0.32	
	AT	109 (0.06)	145 (0.07)	0.87	[0.67-1.12]	0.27					
	AA	2 (<0.01)	2 (<0.01)	1.34	[0.19-9.58]	0.77					
-362*	GG	672 (0.34)	654 (0.28)	1				0.83	[0.76-0.91]	**0.000026**	**0.00021**
	CG	922 (0.47)	1133 (0.49)	0.80	[0.69-0.92]	**0.001**	**0.008**				
	GG	374 (0.19)	514 (0.22)	0.70	[0.59-0.83]	**0.00005**	**0.0004**				
Int1:96	CC	145 (0.09)	152 (0.07)	1				0.87	[0.78-0.96]	0.006	
	CG	677 (0.40)	788 (0.37)	0.90	[0.70-1.16]	0.4					
	GG	862 (0.51)	1170 (0.55)	0.77	[0.60-0.98]	0.037					
Int1:554	WW	743 (0.37)	734 (0.31)	1				0.84	[0.77-0.92]	**0.00014**	**0.00098**
-567	DW	946 (0.47)	1178 (0.50)	0.80	[0.70-0.91]	**0.0009**	**0.0063**				
	DD	320 (0.16)	425 (0.18)	0.73	[0.60-0.98]	**0.0006**	**0.0042**				
+1542	CC	1358 (0.68)	1494 (0.64)	1				0.86	[0.77-0.96]	0.009	
	CT	575 (0.29)	757 (0.32)	0.84	[0.73-0.95]	0.007					
	TT	68 (0.03)	88 (0.04)	0.84	[0.60-1.16]	0.29					
+2413	GG	813 (0.86)	1232 (0.86)	1				1.02	[0.92-1.29]	0.819	
	GT	124 (0.13)	188 (0.13)	0.99	[0.78-1.28]	0.99					
	TT	5 (<0.01)	5 (<0.01)	1.54	[0.44-5-47]	0.5					
+2580	AA	26 (0.01)	21 (<0.01)	1				0.97	[0.84-1.12]	0.711	
	AT	345 (0.17)	412 (0.18)	0.69	[0.38-1.25]	0.22					
	TT	1623 (0.81)	1886 (0.81	0.70	[0.39-1.25]	0.23					

GT, genotype; Int1, intron 1; D, deletion; W, wildtype; OR, odds ratio; CI, 95% confidence interval. P values are adjusted for age, gender and ethnicity. P_nom_, nominal P value; P_corr_, P value after Bonferroni-Holm correction. OR_trend_, estimates of an additive genetic model; *Variants -2581 and -362 were originally genotyped and described in [14].

Stratification for mycobacterial species (*M. tuberculosis vs. M. africanum*) and phylogenetic lineages did not reveal any differences in the associations. Thus, possible confounding exerted by mycobacterial species or distinct genotypes was excluded.

### Haplotypes

We focused on haplotypic combinations comprising the polymorphisms genotyped in our previous study, *MCP1 *-2581A/G and -362C/G, and the deletion or wildtype (W) at intron 1 positions 554-567 (int1del554-567/W), because the variant alleles at these positions are in LD and associations of these variants are presented here and have been described previously [4,6,7,14]. As the combination -2581A/-362G/W occurred as the most frequent haplotype in our study population (frequency [*f*] = 0.55) it was referred to as wildtype reference in further comparisons (Table 4).

**Table 4:** Associations of common haplotypes compared with a reference haplotype

Haplotype			cases	controls	OR	CI	P
-2582	-362	Del	n (frequency)	n (frequency)			
A	G	W	2281 (0.57)	2456 (0.52)	1		
G	C	D	677 (0.17)	928 (0.20)	0.78	[0.69-0.87]	**0.00002**
A	C	D	881 (0.22)	1089 (0.23	0.87	[0.78-0.96]	**0.008**
A	C	W	142 (0.04)	179 (0.04)	0.84	[0.67-1.07]	0.15

Del, intronic 14 bp deletion int1del554-567 OR, odds ratio; CI, 95% confidence interval.

OR and P values refer to comparisons with the reference haplotype A/G/W.

W, wildtype; D, deletion; global adjusted P value 0.0028

The haplotype combinations -2581G/-362C/int1del554-567 and -2581A/-362C/int1del554-567 were significantly associated with resistance to TB compared to the reference haplotype -2581A/-362G/W (OR = 0.78, CI 0.69-0.87, P = 0.00002 and OR = 0.87, CI 0.78-0.96, P = 0.008, respectively; Table 4, Figure 1). The global P value, adjusted through 10 000 permutations, was P_global/adjusted _= 0.0028.

### Reporter gene assay

**Figure 2:**
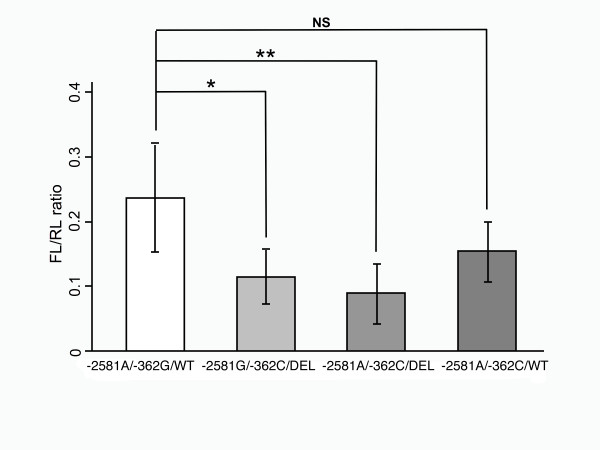
**Histogram illustrating the Firefly/Renilla (FL/RL) ratios**. Histogram illustrating the Firefly/Renilla (FL/RL) ratios obtained after transfection of the four constructs; *, P < 0.05; **, P < 0.01; NS, not significant.

In order to test variant *MCP1 *haplotypes with regard to their impact on gene expression, a luciferase reporter gene assay was performed. Figure 2 shows the plots of the Firefly Luciferase/Renilla Luciferase ratios (FL/RL ratios) that were obtained for the constructs subjected to the assay. An overall ANOVA statistics revealed a significant difference between the FL/RL ratios (P = 0.0019). The calculated studentized range critical value in the *post hoc *pairwise comparisons for variable groups (Tukey-HSD test) was 4.03, and comparisons of the construct carrying the -2581A/-362G/W alleles with the -2581G/-362C/int1del554-567 and -2581A/-362C/int1del554-567 constructs yielded significant results that were above the studentized range critical value (Tukey-HSD test 4.53 and 5.44, respectively).

The P values of a t-test that was calculated with the haplotype -2581A/-362G/W set as reference are given in Table 5. The constructs -2581G/-362C/int1del554-567 and -2581A/-362C/int1del554-567 expressed the luciferase gene to a significantly lower degree than did the wildtype construct -2581A/-362G/W (P = 0.02 and P = 0.006, respectively).

**Table 5:** Reporter gene assay

Haplotype			M	SD	P
-2582	-362	Del			
A	G	W	0.24	0.12	
G	C	D	0.11	0.07	**0.02**
A	C	D	0.08	0.06	**0.006**
A	C	W	0.15	0.06	0.07

Del, intronic 14 bp deletion int1del554-567; D, deletion int1del554-567; W, wildtype; SD, standard deviation; M, arithmetic mean of Firefly Luciferase/Renilla Luciferase (FL/RL) ratios. SD and P values refer to comparisons with the reference haplotype A/G/W.

## Discussion

We have recently described an association of the *MCP1 *-2581G and -362C alleles with protection against TB in a Ghanaian study group [14]. The variants at these positions were in weak linkage disequilibrium (LD; r^2 ^= 0.27; Figure 1). In contrast to our observation of protection conferred by the *MCP1 *-2581G and -362C alleles, earlier research in study groups from Mexico, Korea, Peru and Zambia has attributed to *MCP1 *-2581G an increased risk to develop clinical TB [4,6,7]. Moreover, *MCP1 *-2581G and -362C were shown to enhance promoter activity *in vitro *in individuals from Korea (*MCP1 *-2581G) and in Caucasians (*MCP1 *-2581G and -362C), respectively [8,9,15], consistent with studies indicating that in pulmonary TB MCP-1 plasma levels are elevated [3,4].

Among the eight variants that were in LD with *MCP1 *-362 and/or *MCP1 *-2581 according to data available from NCBI and therefore subjected to genotyping in the present study we found the 14 base pair deletion in intron 1 (int1del554-567) associated with protection against TB similar to the ORs of *MCP1 *-2581G and *MCP1 *-362C.

As the three variants are in weak LD (*MCP1 *-2581A/G with *MCP1 *-362C/G and *MCP1 *int1del554-567/W) or in strong LD (*MCP1 *-362C/G with *MCP1 *int1del554-567/W) in the study group (Figure 1), haplotypes might explain more adequately than single mutations the genetic association and its relation to MCP-1 production. All haplotypic combination comprising the variants *MCP1 *-2581G/A, *MCP1 *-362C/G and int1del554-567/W and occuring at frequencies (*f*) >0.01 were considered (Table 4). The strongest association of protection against TB was with the haplotypic combination -2581G/-362C/int1del554-567 (*f* = 0.19) compared to the wildtype haplotype -2581A/-362G/W (*f* = 0.54; OR = 0.78, CI 0.69-0.87, P = 0.00002). Carriers of the haplotypic combination -2581A/-362C/int1del554-567 (*f* = 0.23) are slightly less, but still significantly protected against TB (OR = 0.87, CI 0.78-0.96, P = 0.008).

To further examine the influence of haplotypes on the promoter activity, a reporter luciferase assay with constructs comprising the -2581A/G and -362C/G promoter variants, the first exon (149 bp) and the intronic 14 bp deletion int1del554-567 or the wildtype sequence at these positions was performed. Only those combinations that occurred in frequencies >1% in the study population were included. The findings of the reporter gene assay corresponded to the results of the genetic analysis. A significant decrease of gene expression occurred in constructs carrying the -2581G/-362C/int1del554-567 and -2581A/-362C/int1del554-567 combinations (P = 0.02 and P = 0.006, respectively; Table 5). It may be inferred from the results of the reporter gene assay that both variants, *MCP1 *-362C and int1del554-567, exert a reduction of the transcriptional activity, eventually resulting in lowered production of MCP-1.

	Several mechanisms might be involved in the decrease of MCP-1 production. First, *MCP1 *-362G constitutes a binding site for the Signal Transducer and Activator of Transcription 1 (Stat-1) [16]. Stat-1 is a transcription factor that enhances gene expression, and deprivation of Stat-1 binding through a loss of its binding site might reduce gene transcription, as seen in the luciferase assay. Another mechanism for a reduction of transcription is provided by the fact that Intronic deletions often cause a decrease of transcriptional activity [18,19] and influence RNA stability [20]. Lastly, the 14 bp deletion int1del554-567 in the first intron of the *MCP1 *gene causes a loss of a predicted alternative splice site http://zeus2.itb.cnr.it/~webgene/wwwspliceview_ex.html. While transcripts with several alternative splice sites appear to be more robust, a loss of transcription sites could promote random degeneration in the nucleus [21]. It is, therefore, reasonable to ascribe a reduced MCP-1 production to the int1del554-567 deletion.

## Conclusions

In addition to the results of the previous study of the Ghanaian TB case-control sample, we have now identified the haplotype combination -2581G/-362C/int1del554-567 that mediates stronger protection than does the *MCP1 *-362C allele alone (OR = 0.78, CI 0.69-0.87 vs OR = 0.83, CI 0.76-0.91). Our findings in both the genetic analysis and the reporter gene study further indicate a largely negligible role of the variant at position -2581.

The genetic risk of TB observed for variation in the *MCP1 *promoter and in intron 1 is most likely conferred through an alteration of the *MCP1 *expression, in line with the previous findings that increased MCP-1 production favours the occurrence of clinical TB. A similar observation was made in a mouse model for infections with both *Listeria monocytogenes *and *M. tuberculosis*, where increased *MCP1 *expression in transgenic mice led to a 1 to 1.5 log greater sensitivity to infection [22]. It has been described that in *MCP1 *deficient mice subjected to low-dose aerosol infection with *M. tuberculosis *Erdman the number of macrophages that enter the lung is decreased. As a consequence, these mice initially harbour higher bacterial loads in their lungs compared to control animals, but eventually established a stable state of chronic disease [23]. No significant difference to *MCP1 *wildtype mice in the susceptibility to intravenous infection was found [24]. It was also shown that mice that overexpress *MCP1 *in their lungs exhibit increased uptake of *M. tuberculosis *BCG in dendritic cells compared to wildtype animals [25].

To date it remains unclear why high MCP-1 levels cause increased TB susceptibility in humans and how MCP-1 levels interact with the efficiency of the MCP-1 gradient. Pertinent explanations could be that high systemic concentrations of MCP-1 would trigger the desensitization of receptors and reduce signal transduction or might lead to an adjustment and, as a consequence, to the neutralization of the chemoattractant gradient that is required to escort sensitive monocytes to the sites of tissue damage.

## Methods

### Study group

The study design and the enrollment procedure have been described in detail previously [14,26]. In brief, participants were recruited at the two major Ghanaian teaching hospitals in Accra and Kumasi and at additional hospitals and polyclinics in these metropolitan areas and at regional district hospitals. 2010 HIV-negative individuals with smear- and/or culture-positive pulmonary TB were recruited as cases. The control group consisted of 2346 individuals, from whom 1211 were unrelated personal contacts of cases and 1135 were community members from the adjacent neighbourhood or working contacts. The proportion of ethnic groups did not differ significantly between cases and controls. Participants belonged to the ethnic groups of Akan, Ga-Adangbe, Ewe and groups from northern Ghana, including Dagomba, Sissala, Gonja and Kusasi. The male-to-female ratio in the total study group was 1:0.58, and the mean age of participants was 33 years without gender differences. The characterization of phenotypes included documentation of the medical history of cases on structured questionnaires, two independent examinations of non-induced sputum specimens, serological determination of the HIV status, culturing and molecular differentiation of phylogenetic lineages of mycobacterial clades and posterior-anterior chest X-rays. Positive HIV test results were verified in an alternate test system. Fine-typing of genotypes by spoligotyping, *IS*6110 fingerprinting and determination of drug resistance was performed as previously described [14]. TB-patients were included for specific treatment in the DOTS programme (Directly Observed Treatment Short-Course strategy) organized by the Ghanaian National Tuberculosis Programme.

Of the control group, the medical history was obtained and a clinical examination was performed. Chest X-rays did not reveal any signs of actual or past pulmonary TB. In addition, a tuberculin skin test (TST, Tuberculin Test PPD Mérieux, bioMérieux, Nürtingen, Germany) was performed. The TST was positive in 2217 controls and 129 control individuals were TST-negative.

Ethical approval of the study design was obtained by the Committee on Human Research, Publications and Ethics, College of Health Sciences, Kwame Nkrumah University of Science and Technology, Kumasi, Ghana, and the Ethics Committee of the Ghana Health Service, Accra, Ghana. Informed consent was given by study participants either by signature or, in case of illiteracy, by thumbprint in the presence of a witness. The aims of the study and the procedure of venous blood collection were explained before blood samples were taken.

### Variants selected for genotyping; genetic analyses

According to the most recent data of the haplotype structure of *MCP1 *obtained from the innate immunity database https://pharmgat.org/IIPGA2/PGAs/InnateImmunity/CCL2/ we selected eight *MCP1 *polymorphisms that are in LD with the *MCP1 *-362 promoter variant which has previously shown the strongest association [14].

Table 1 lists the variants that were selected, including their rs numbers and PCR amplification primers as well as sensor/anchor nucleotides for LightTyper-based genotyping. Three variants are located in the promoter region, two in the first intron and three in the 3'-UTR.

Standard methods were applied to extract DNA from full venous blood and genotypes of the *MCP1 *variants were determined by fluorescence resonance energy transfer (LightTyper^®^; Roche Diagnostics, Mannheim, Germany) with dynamic allele specific hybridization.

### Databases and statistical analyses

Demographic and self reported data was double entered into a Fourth Dimension database (San José, CA, USA). Genotype frequencies and odds ratios as well as Hardy-Weinberg equilibria (HWE) were calculated with the Stata 10 software (Stata Corporation, College Station, TX, USA) and logistic regression was applied to adjust for age, gender and ethnicities. Allelic and haplotype frequencies and associations were used to reconstruct haplotypes, calculated with the Unphased software (version 3.1.4; http://www.mrc-bsu.cam.ac.uk/personal/frank/software/unphased). P values were adjusted through 10 000 permutations. Haploview version 4.1 http://www.broad.mit.edu/mpg/haploview/ was used to calculate linkage disequilibria (LD, given as r^2^) and to generate the graphical output. The Tukey Honestly Significant Difference test (Tukey-HSD test) was performed for *post hoc *comparisons of variable groups in the evaluation of the reporter gene assay.

The power to determine a genetic effect (CaTS software; http://www.sph.umich.edu/csg/abecasis/CaTS/) with a genotype relative risk of 1.4 was, with 2010 cases and 2346 controls and assuming a disease allele frequency of 0.2, a prevalence 0.003 and a significance level of 1 × 10^-7 ^was 89%.

### Reporter gene assay, engineering of constructs and transfection

The PGL2-Control Vector (Promega, Mannheim, Germany) was used for cloning of all constructs of interest. Four fragments of the *MCP1 *gene, each of 3569 bp length and containing the promoter, the first exon and the first intron, were PCR-amplified with primers 5'- caccaagaggagcttttcca-3' and 5'-gcgcacgcgtcctctgcactgagatcttcct-3'. The *MCP1 *-2581A/G and -362C/G variants as well as the deletion int1del554-567/W were examined. Only haplotype combinations occurring with frequencies >1% were subjected to the reporter gene assay. The following combinations were included: *MCP1 *-2581A/-362G/W; -2581G/-362C/int1del554-567; -2581A/-362C/int1del554-567; -2581A/-362C/W. The Expand Long Template PCR System (Roche, Mannheim, Germany) was used for PCR-amplification.

PCR conditions were: Initial denaturation (94°C, 2 min), 10 amplification cycles (98°C, 10'; 60°C, 30'; 68°C, 10''), 25 amplification cycles (98°C, 15'; 62°C, 30'; 68°C, 20'') and final elongation (78°C, 7''). SMA1 and MLU1 restriction sites at the 5' and 3'ends, respectively, were engineered on each PCR product. In an intermediate step, the fragments were gel-purified, ligated into a pCR-XL-TOPO plasmid (Invitrogen, Carlsbad, USA) and subsequently transfected into Top10 cells (Invitrogen, Carlsbad, USA) according to the manufacturer's instructions. After overnight incubation, cells were lysated and plasmids were digested with SMA1 and MLU1 restriction enzymes (New England Biolabs, Ipswich, USA). The resulting fragments were then ligated into the PGL2 Cloning Vector and transfected into Top10 cells. The final constructs were isolated using an EndoFree Plasmid Maxi Kit (Qiagen, Hilden, Germany).

The Bio-RAD Gene Pulser Xcell system (Bio-Rad Laboratories Ltd., Hertfordshire, UK) was used for co-transfection of 6 × 10^6 ^THP1-cells (German Resource Centre for Biological Material, DSMZ [Deutsche Sammlung für Mikroorganismen und Zellkulturen], Braunschweig, Germany) with 0,5 μg of the phRL-CMV vector and either 0,5 μg of the pGL2-Control vector or 0,5 μg of one of the four plasmid constructs. Four hours after transfection, cells were harvested and luciferase activities were measured using a single tube Junior LB9509 luminometer (Berthold Technologies, Bad Wildbad, Germany) and the Dual-Luciferase Reporter Assay System (Promega, Mannheim, Germany). After a 10 second period of Firefly luminescence measurement, 100 ml 1× Stop & Glo Reagent that is supplied with the Dual-Luciferase Reporter Assay System kit were added and Renilla luminescence was detected in another 10 second measurement period. Ten independent transfections and measurements were performed for each construct.

## Competing interests

The authors declare that they have no competing interests.

## Authors' contributions

CDI, TT, RDH and CGM conceived and designed the experiments. CDI and BF performed the experiments. CDI, TT and CGM analyzed the data. CGM wrote the paper. EOD supervised the sample collection in Ghana. JOG and EOD designed the study and performed the phenotyping of patients and controls. All authors read and approved the final manuscript.
